# 8-Substituted Triazolobenzodiazepines: *In Vitro* and *In Vivo* Pharmacology in Relation to Structural Docking at the α1 Subunit-Containing GABA_A_ Receptor

**DOI:** 10.3389/fphar.2021.625233

**Published:** 2021-04-20

**Authors:** Lalit K. Golani, Donna M. Platt, Daniela Rüedi-Bettschen, Chitra Edwanker, Shenming Huang, Michael M. Poe, Roman Furtmüller, Werner Sieghart, James M. Cook, James K. Rowlett

**Affiliations:** ^1^ Department of Chemistry and Biochemistry, University of Wisconsin-Milwaukee, Milwaukee, WI, United States; ^2^ Department of Psychiatry and Human Behavior, University of Mississippi Medical Center, Jackson, MS, United States; ^3^ Harvard Medical School, New England Primate Research Center, Southborough, MA, United States; ^4^ Brain Research Institute, Medical University, Vienna, Austria

**Keywords:** benzodiazepine, anxiolytic, sedation, squirrel monkey, rhesus monkey

## Abstract

In order to develop improved anxiolytic drugs, 8-substituted analogs of triazolam were synthesized in an effort to discover compounds with selectivity for α2/α3 subunit-containing GABA_A_ subtypes. Two compounds in this series, XLi-JY-DMH (6-(2-chlorophenyl)-8-ethynyl-1-methyl-4H-benzo [f][1,2,4]triazolo[4,3-a][1,4]diazepine) and SH-TRI-108 [(E)-8-ethynyl-1-methyl-6-(pyridin-2-yl)-4H-benzo [f][1,2,4]triazolo[4,3-a][1,4]diazepine], were evaluated for *in vitro* and *in vivo* properties associated with GABA_A_ subtype-selective ligands. In radioligand binding assays conducted in transfected HEK cells containing rat αXβ3γ2 subtypes (X = 1,2,3,5), no evidence of selectivity was obtained, although differences in potency relative to triazolam were observed overall (triazolam > XLi-JY-DMH > SH-TRI-108). In studies with rat αXβ3γ2 subtypes (X = 1,2,3,5) using patch-clamp electrophysiology, no differences in maximal potentiation of GABA-mediated Cl^−^ current was obtained across subtypes for any compound. However, SH-TRI-108 demonstrated a 25-fold difference in functional potency between α1β3γ2 vs. α2β3γ2 subtypes. We evaluated the extent to which this potency difference translated into behavioral pharmacological differences in monkeys. In a rhesus monkey conflict model of anxiolytic-like effects, triazolam, XLi-JY-DMH, and SH-TR-108 increased rates of responding attenuated by shock (anti-conflict effect) but also attenuated non-suppressed responding. In a squirrel monkey observation procedure, both analogs engendered a profile of sedative-motor effects similar to that reported previously for triazolam. In molecular docking studies, we found that the interactions of the 8-ethynyl triazolobenzodiazepines with the C-loop of the α1GABA_A_ site was stronger than that of imidazodiazepines XHe-II-053 and HZ-166, which may account for the non-sedating yet anxiolytic profile of these latter compounds when evaluated in previous studies.

## Introduction

Anxiolytic and sedative benzodiazepines (BZs) produce their pharmacological effects by enhancing the inhibitory action of γ-aminobutyric acid (GABA) at type A GABA (GABA_A_) receptors throughout the brain. The GABA_A_ receptor is a pentameric chloride ionophore composed of subunits from at least five different families; the α, ß and γ subunits are necessary to confer sensitivity to BZs (for reviews, Möhler, 2011; Engin et al., 2018). Conventional BZs (e.g., triazolam) bind non-selectively to GABA_A_ receptors that contain α1, α2, α3, and α5 subunits (α1GABA_A_, α2GABA_A_, α3GABA_A_, and α5GABA_A_ receptors, respectively) while they do not bind appreciably to α4-and α6 subunit-containing GABA_A_ receptors. This binding profile may be responsible for the myriad of behavioral effects produced by BZs, including a role for α1GABA_A_ receptors in the anticonvulsant, sedative, and motor effects of BZs, a role for α2GABA_A_ and α3GABA_A_ receptors in the anxiolytic, anticonvulsant, antinociceptive, and myorelaxant effects of BZs, and a role for α5GABA_A_ receptors in BZ-associated memory processes as well as anxiolytic effects (e.g., Witkin et al., 2017; Witklin et al., 2018; Witkin et al., 2020; Tudeau et al., 2020 for review, Engin et al., 2018).

Recent research efforts have been directed at developing compounds that are “functionally selective” in that they may bind to all four BZ-sensitive subunits of the GABA_A_ receptor, but have intrinsic efficacy at only the desired GABA_A_ receptor subtypes. This functional subtype-selectivity framework has been used to develop anxiolytics that potentially have reduced side effects. Because the BZ scaffold historically has proven to be generally nontoxic with desirable pharmacokinetic properties, one approach to developing functionally selective compounds has been to identify and advance a selected group of 8-substituted triazolobenzodiazepines and imidazodiazepines (e.g., Rivas et al., 2009; Poe et al., 2016). The focus recently has been on bioisostere imidazodiazepines in a novel series of compounds with preferential intrinsic efficacy at α2/3GABA_A_ subtypes (Poe et al., 2016; Witkin et al., 2017; Witkin et al., 2018; Witkin et al., 2020), which demonstrated robust anticonvulsant properties, anxiolytic-like effects, antinociceptive effects, but with a reduced propensity to engender sedative-motor disruptions. The present study reports data from key compounds from a series of 8-substituted analogs of triazolam. Triazolam is noteworthy for having high affinity for GABA_A_ subtypes, albeit without selectivity, along with the additional advantage of limited active metabolites (e.g., Ducić et al., 1993). One goal of our studies was to assess the extent to which the favorable *in vitro* and *in vivo* profile of imidazodiazepines may be extended to the 8-substituted triazolobenzodiazepine series.

**FIGURE 1:**
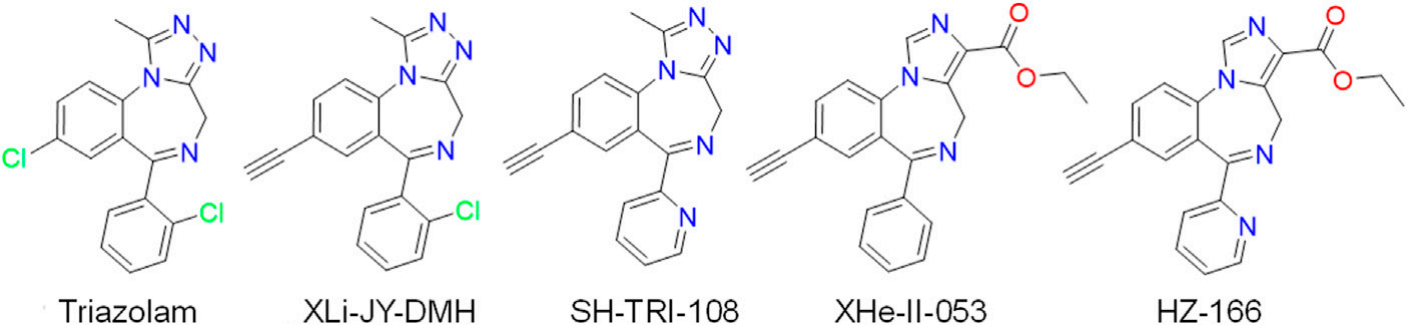
Chemical structures of triazolam, 8-substituted triazolobenzodiazepines (XLi-JY-DMH, SH-TRI-108) and 8-substituted imidazodiazepines (XHe-II-053, HZ-166).

The triazolobenzodiazepine triazolam (Halcion®, as well as the analog, alprazolam, Xanax®) is a potent sedative-anxiolytic drug in clinical use. The present studies describe the binding affinity and efficacy profiles of two analogs of triazolam: XLi-JY-DMH (6-(2-chlorophenyl)-8-ethynyl-1-methyl-4H-benzo [f][1,2,4]triazolo[4,3-a][1,4]diazepine) and SH-TRI-108 [(E)-8-ethynyl-1-methyl-6-(pyridin-2-yl)-4H-benzo [f][1,2,4]triazolo[4,3-a][1,4]diazepine], two compounds that have been developed as part of the 8-substituted triazolobenzodiazepine series (Figure 1). As part of our anxiolytic development program, we conducted tests in rhesus monkeys trained in a conflict model of anxiolytic-like effects, which has reliably shown predictive validity for relative potencies for behavioral effects in human subjects (Rowlett et al., 2006). In addition, sedative-motor effects were evaluated using observation and hands-on techniques in squirrel monkeys (Platt et al., 2002; Licata et al., 2009). Finally, to provide mechanistic information regarding the results with triazolobenzodiazepines in comparison with imidazodiazepines, we conducted molecular modeling experiments with compounds evaluated in this study in comparison to representative imidazodiazepines (Figure 1).

## Methods

### Radioligand Binding Assay

#### Culture and Transfection of HEK 293 Cells

Transformed human embryonic kidney (HEK 293) cells were cultured, transfected and harvested as described in Pöltl et al. (2003). Briefly, transformed HEK 293 cells (CRL 1573; American Type Culture Collection, Rockville, MD) were cultured in Dulbecco’s modified Eagle’s medium supplemented with 10% fetal calf serum, 2 mM l-glutamine, 50 μM ß mercaptoethanol, 100 U/ml Penicillin G, 100 μg/ml streptomycin and 1% MEM (Minimal Essential Medium = non essential amino acids) in 75 cm^2^ Petri dishes by the use of standard cell culture techniques. HEK 293 cells were transfected with a total of 21 μg of subunit cDNA (rat: α1, α2, α3, α5, β3, γ2; cloned in pCI; Promega, Madison, WI) at a cDNA ratio of αx: βx: γx = 1:1:1 via the calcium phosphate precipitation method as described by Chen and Okayama (1988). This cDNA ratio has been used previously for the expression of various GABA_A_ receptors in [^3^H]muscimol or [^3^H]Ro 15–1788 binding studies (e.g., Zezula et al., 1996). The cells were harvested in phosphate-buffered saline (PBS; 137 mM NaCl, 2.7 mM KCl, 1.5 mM KH_2_PO_4_, 4.3 mM Na_2_HPO_4_ pH 7.3) 48 h after transfection.

#### Radioligand Binding

For inhibition studies, frozen membranes from transfected HEK cells were thawed, homogenized in 50 mM Tris/citrate buffer (pH 7.4) using an Ultra-Turrax, and then followed by two centrifugation resuspension cycles (200,000 x g for 20 min at 4°C). Cell pellets were resuspended in 50 mM Tris/citrate buffer (pH 7.4) at a protein concentration in the range of 0.1–1 mg/ml as measured with the BCA protein assay kit (Pierce, Rockford, IL) using bovine serum albumin as standard.

Three hundred (300) μL of the final homogenates were suspended in 1 ml of a solution containing 50 mM Tris/citrate buffer (pH 7.1), 150 mM NaCl, 2 nM [^3^H]flunitrazepam (74.1 Ci/mmol) in the absence or presence of 100 μM flunitrazepam, 10 pM–10 μM triazolam, 1 nM–300 nM XLi-JY-DMH, or 3 nM–1 μM SH-TRI-108. After incubation for 90 min at 4°C, the suspensions were rapidly filtered through Whatman GF/B filters using a multi-channel receptor binding harvester (Brandel Inc., Gaithersburg, MD), washed three times with 3 ml of 50 mM Tris/citrate buffer (pH 7.1) and subjected to liquid scintillation counting (Filter-Count, Packard; 2100 TR Tri-Carb Scintillation Analyser, Packard). Data were analyzed using GraphPad Prism (Graph Pad Software Inc., San Diego, CA). K_i_ values were calculated using the Cheng Prusoff equation (Cheng and Prusoff, 1973).

### Two Electrode Voltage Clamp Assay

#### Preparation of Cloned mRNA

Cloning of rat GABA_A_ receptor subunits α1, β3 and γ2 into pCDM8 expression vectors (Invitrogen Co.; Carlsbad, CA) has been described elsewhere (Fuchs et al., 1995). cDNAs for subunits α2, α3 and α5 were gifts from P. Malherbe and were subcloned into the pCI-vector. After linearizing the cDNA vectors with appropriate restriction endonucleases, capped transcripts were produced using the mMessage mMachine T7 transcription kit (Ambion, Inc.; Austin, TX). The capped transcripts were polyadenylated using yeast poly(A) polymerase (USB; Cleveland, OH) and were diluted and stored in diethylpyrocarbonate-treated water at –70°C.

#### Functional Expression of GABA_A_ Receptors

The methods used for isolating, culturing, injecting and defolliculating of oocytes are identical to those described by Sigel and colleagues (Sigel, 1987; Sigel et al., 1990). Mature female *Xenopus laevis* (Nasco, Inc.; Fort Atkinson, WI) were anesthetized in a bath of ice-cold 0.17% Tricain (Ethyl-m-aminobenzoat) before decapitation and removal of the frog ovaries. Stage 5–6 oocytes with the follicle cell layer around them were singled out of the ovary using a platinum wire loop. Oocytes were stored and incubated at 18°C in modified Barths’ Medium [MB, containing 88 mM NaCl, 10 mM HEPES-NaOH (pH 7.4), 2.4 mM NaHCO_3_, 1 mM KCl, 0.82 mM MgSO_4_, 0.41 mM CaCl_2_, 0.34 mM Ca(NO_3_)_2_] that was supplemented with 100 U/ml penicillin and 100 μg/ml streptomycin. Oocytes with follicle cell layers still around them were injected with 50 nL of an aqueous solution of cRNA. This solution contained 0.0065 ng/nL of the transcripts for the α and the β3 subunit, as well as 0.032 ng/nL of the transcript for the γ2 subunit. *Xenopus* oocytes were injected with rat α1β3γ2, α2β3γ2, α3β3γ2, or α5β3γ2 subunit combinations.

After injection of cRNA, oocytes were incubated for at least 36 h before the enveloping follicle cell layers were removed. To this end, oocytes were incubated for 20 min at 37°C in MB that contained 1 mg/ml collagenase type IA and 0.1 mg/ml trypsin inhibitor I-S. This was followed by osmotic shrinkage of the oocytes in doubly concentrated MB medium supplied with 4 mM Na-EGTA. Finally, the oocytes were transferred to a culture dish containing MB and were gently pushed away from the follicle cell layer which stuck to the surface of the dish. After removing the follicle cell layer, oocytes were allowed to recover for at least 4 h before being used in electrophysiological experiments.

#### Electrophysiological Recording

Oocytes were placed on a nylon-grid in a bath of *Xenopus* Ringer solution [XR, containing 90 mM NaCl, 5 mM HEPES-NaOH (pH 7.4), 1 mM MgCl_2_, 1 mM KCl and 1 mM CaCl_2_]. The oocytes were constantly washed by a flow of 6 ml/min XR which could be switched to XR containing GABA and/or drugs. Drugs were diluted into XR from DMSO-solutions resulting in a final concentration of 0.1% DMSO perfusing the oocytes. Drugs were preapplied for 30 s before the addition of GABA (concentration equal to EC_3_), which was then coapplied with the drugs until a peak response was observed. Between two applications, oocytes were washed in XR for up to 15 min to ensure full recovery from desensitization. For current measurements, the oocytes were impaled with two microelectrodes (2–3 mΩ) which were filled with 2 mM KCl. All recordings were performed at room temperature at a holding potential of –60 mV using a Warner OC-725C two-electrode voltage clamp (Warner Instruments; Hamden, CT) or a Dagan CA-1B Oocyte Clamp (Dagan Co.; Minneapolis, MN). Data were digitized, recorded and measured using a Digidata 1322 A data acquisition system (Axon Instruments; Union City, CA).

#### Data Analyses

Results of concentration response experiments were fitted using GraphPad Prism 3.00. The equation used for fitting concentration response curves was Y=Bottom + (Top-Bottom)/[1 + 10^([LogEC_50_-X] * nH)]. X represents the logarithm of concentration, Y represents the response, and nH represents the Hill Slope. Y starts at Bottom and goes to Top with a sigmoid shape. This is identical to the “four parameter logistic equation”. This iterative curve-fitting approach was used to obtain EC_50_, maximum effect, and Hill slope parameters based on the percent increase in Cl^−^ current induced by GABA application.

The curve-fitting approach also was used in an additional analysis, based on converting the “raw” data to a ratio with the results from triazolam. The rationale for this analysis was that the two analogs were evaluated at the same concentrations as triazolam. Because triazolam is a well-characterized BZ ligand *in vitro* and *in vivo*, we could then derive both potency and maximum effect data as “relative to triazolam”, which is considered to be a non-selective ligand. Therefore, assuming comparable receptor engagement, the behavioral data was interpreted as differences due to the extent that XLi-JY-DMH and/or SH-TRI-108 differed in potency and/or efficacy at receptor subtypes, relative to triazolam.

### Conflict Procedure

#### Subjects and Surgical Procedure

Three adult male rhesus monkeys (*Macaca mulatta*), weighing 6.3–10.3 kg, were studied in daily experimental sessions (Monday to Friday). Between sessions, monkeys lived in individual home cages where they had unlimited access to water. The monkeys were maintained at ∼90% of their free-feeding body weight by adjusting their access to food in the home cage (Teklad Monkey Diet supplemented with fresh fruit). All animals were maintained in accordance with the guidelines of the Committee on Animals of the Harvard Medical School and the “Guide for Care and Use of Laboratory Animals” of the Institute of Laboratory Animal Resources, National Research Council, Department of Health, Education and Welfare Publication No (NIH) 85–23, revised 1996. Research protocols were approved by the Harvard Medical School Institutional Animal Care and Use Committee.

Monkeys were prepared with chronic indwelling venous catheters (polyvinyl chloride, i.d.: 0.64 mm; o. d.: 1.35 mm) using the general surgical procedures described by Platt et al. (2005). Under isoflurane anesthesia and aseptic conditions, one end of a catheter was passed to the level of the right atrium by way of a brachial, femoral or jugular vein. The distal end of the catheter was passed subcutaneously and exited in the mid-scapular region. Catheters were flushed daily with heparinized saline (150–200 U/ml) and were sealed with stainless steel obturators when not in use. Monkeys wore custom-made nylon-mesh jackets (Lomir Biomedical, Toronto, Canada) at all times to protect the catheter.

#### Apparatus

Experimental sessions were conducted in ventilated and sound-attenuating chambers. Monkeys were seated in custom-made primate chairs (Crist Instrument Co., Hagerstown, MD). One response lever (model ENV-610M, Med Associates, Inc., Georgia, VT) was mounted on the wall of the chamber in front of the monkey. Each press of a lever with a minimum downward force of approximately 0.25 N produced an audible click and was recorded as a response. Food pellets (Formula 05474, 1 g, Bioserve, Frenchtown, NJ) could be delivered to a tray located between the levers. Colored lights mounted above the levers could be illuminated to serve as visual stimuli. Child-size shoes were fitted with brass electrode plates and were connected to a shock generator (Med Associates; Georgia, VT). Electrode gel (Parker Laboratories, Inc.; Fairfield, NJ) was applied to the plates to facilitate contact between the plates and the monkeys’ feet.

#### Procedure

Monkeys were trained to respond under a multiple schedule of food reinforcement consisting of two components: 1) a schedule of food delivery only, and 2) a schedule of food pellet delivery plus a schedule of foot shock delivery (0.25 s duration, 1–3 mA depending on the individual monkey). Four components were available in a session, separated by 10 min time out periods in which responding had no programmed consequences. Responding was maintained in each component under an 18-response, fixed-ratio (FR18) schedule of food pellet delivery. Each component consisted of the schedule of food pellet delivery signaled by red stimulus lights, followed immediately by the same schedule of food delivery combined with a FR20 schedule of foot shock delivery, signaled by green stimulus lights. Each delivery of a food pellet was followed by a 10 s time out. Drugs were administered during the 5th min of the 10 min time out that preceded each component.

Training sessions were conducted 5 days/week until performance in both food only and food + shock components was stable (i.e., no upward or downward trends in rates of responding for 3 consecutive days). In addition, if rates of responding in a component during a training session were greater or less than 20% of the corresponding response rates in the previous training session, additional training sessions were conducted until responding was again stable. Once training criteria were met, test sessions were conducted once or twice per week, separated by at least two days. Dose-response functions were determined for XLi-JY-DMH (0.003–0.3 mg/kg), SH-TRI-108 (0.03–1.8 mg/kg), as well as the non-selective BZ agonist triazolam (0.0003–0.03 mg/kg), using a cumulative dosing procedure similar to the one described by Rowlett et al. (2005). Four-point cumulative dose-response functions were determined within a single test session by administering incremental doses (½ log units) of drug i.v. during time out periods that preceded sequential components. Five or more different doses of a drug were studied by administering overlapping ranges of cumulative doses during test sessions on different days. All monkeys in this study received all compounds and all doses of each compound.

#### Data Analysis

The number of responses in a component, minus responding during pellet delivery and the 10-s time outs, was divided by the total component time minus the 10 s time outs to obtain rates of responding (responses). Data for multiple determinations were averaged for an individual monkey, and these response rates were averaged across monkeys (mean responses/s ± SEM). To determine statistical reliability of treatment effects on responding during food only and food + shock components, the effect of dose was determined for each drug by separate repeated measures ANOVAs. Treatment effects were assessed further using the Fisher’s LSD method, in which the comparison of interest was the average response rate engendered by each dose of test drug vs. the average response rate after vehicle administration (alpha level = *p* ≤ 0.05). Effect sizes were calculated according to the approach of Keppel and Wickens (2004).

### Observable Behavior

#### Subjects

Eight adult male squirrel monkeys (*Saimiri sciureus*), weighing 750–1,100 g, were studied in daily experimental sessions (Monday to Friday). Between sessions, monkeys lived in individual home cages where they had unrestricted access to food (Teklad Monkey Diet supplemented with fresh fruit) and water. All animals were maintained in accordance with the guidelines of the Committee on Animals of the Harvard Medical School and the “Guide for Care and Use of Laboratory Animals” of the Institute of Laboratory Animal Resources, National Research Council, Department of Health, Education and Welfare Publication No (NIH) 85–23, revised 1996. Research protocols were approved by the Harvard Medical School Institutional Animal Care and Use Committee.

#### Apparatus and Procedure

Studies were conducted in a ventilated, transparent Plexiglas arena (114 cm × 122 cm X 213 cm) located in a room that was isolated from other animals. The arena was equipped with perches, plastic chains suspended from the ceiling, and a wood chip foraging substrate to provide opportunities for monkeys to express a range of species-typical motor behaviors. A video camera was positioned approximately 1 m in front of the chamber and operated continuously during the observation session.

The monkeys initially were habituated to the observation arena and the handling and injection procedures described below for a period of approximately one month. Following habituation, 30 min observation sessions were conducted daily, during which the animal’s behavior was videotaped continuously. This procedure provided an archival record of experimental sessions and permitted subsequent scoring of videotapes by independent observers. Ataxia and muscle relaxation were measured in the same animals after the sixth, eighteenth and thirtieth min of each 30 min session. The monkeys were removed briefly from the observation arena by a trained handler and evaluated for ataxia, defined as the inability to balance on a 1 cm diameter stainless steel pole held in the horizontal plane. A score of 0 indicated that the monkey was able to balance normally on the pole, a score of 1 indicated that the monkey was able to hold on to the pole but unable to maintain balance (e.g., hang suspended by limbs below pole), and a score of 2 indicated that the monkey could neither balance on nor hold on to the pole. Muscle relaxation was defined as decreased resistance to extension of a hind limb. During each evaluation, a score of 0 indicated normal resistance to hind limb extension, a score of (-1) indicated decreased resistance to hind limb extension, and a score of (-2) indicated no resistance to hind limb extension (i.e., the monkey was flaccid and completely relaxed).

After the habituation period described above, drug tests were conducted once or twice per week, with control sessions preceded by saline injections on intervening days. Various doses of XLi-JY-DMH (0.01–1.0 mg/kg) and SH-TRI-108 (0.03–3.0 mg/kg) were evaluated in separate test sessions. The presession injection time of 30 min was selected on the basis of preliminary experiments to determine approximate time to peak effect for each drug. All drugs, as well as saline controls, were administered i. m. in a calf or thigh muscle.

Scoring of videotapes was conducted by three observers trained to use the behavioral scoring system described by Platt et al. (2002). The observers were not informed about the drugs under investigation. To assure reliability across observers, each underwent at least 20 h of training until they reached an inter-observer reliability criterion of ≥90% based on percent agreement scores calculated between all possible pairs. The behavioral scoring system included 8 categories (Table 1), which were scored by recording the presence or absence of each behavior in 15 s intervals during three 5 min observation periods across the session (0–5 min, 12–17 min, 24–29 min). Scores were calculated from these data as the number of intervals in which a particular behavior occurred. The maximum possible score was 20. To facilitate data analysis, object exploration and foraging were combined into the more general category of environment-directed behavior, and self-grooming and scratching were combined into the more general category of self-directed behavior.

**TABLE 1:** Behavioral definitions.

**Locomotion** – any two or more directed steps in the horizontal and/or vertical plane
**Environment-directed**
** Object exploration** – any tactile or oral manipulation of features of the observational arena
** Foraging** – sweeping and/or picking through wood chip substrate
**Self-directed**
** Self-grooming** – picking, scraping, spreading or licking of fur
** Scratching** – movement of digits through fur in a rhythmic, repeated motion
**Rest posture** – species-typical position: Head tucked to chest, tail wrapped around upper body
**Procumbent posture** – loose-limbed, sprawled, unable to maintain an upright position, lying on floor
**Other** – any notable behavior not defined above (e.g., yawn, sneeze)

A separate group of squirrel monkeys (N = 4) were used for the food pellet consumption study. These monkeys were placed in the observation chamber and habituated to the chamber as described above. For these studies, the observation chamber was modified such that a stainless steel food bowl could be secured during a session. To establish baseline levels of food pellet consumption, each monkey was administered a saline injection (i.m.) 5 min before being placed in the observation chamber, and was given access to 100 nutritionally appropriate sucrose pellets (Formula F/Fp, sucrose with fruit punch, 190 mg, Noyes Precision Food Pellets, Lancaster, NH, USA) for 10 min. Drug test sessions were conducted two to three times per week with saline control sessions on intervening days. At the end of the 10 min period, the monkey was removed and the remaining pellets counted, subtracted from 100, and recorded as pellets consumed”.

#### Data Analysis

Data obtained from the videotapes and sucrose pellets consumed were analyzed using parametric statistics, after analyses using Shapiro-Wilks tests determined the data conformed to normal distributions (*p*’s ≤ 0.05). For the pellets consumed data, the data were converted to percent of individual monkey’s baselines obtained from the intervening days of drug test sessions. For each subject, scores for each behavior were averaged across the three observation periods of a session because no reliable differences were identified by separate repeated measures ANOVAs. Scores were then averaged across subjects to obtain group means. To determine statistical reliability of treatment effects on each behavior, the effect of dose was determined for each drug by separate repeated measures ANOVAs. Treatment effects were assessed further using Fisher’s LSD method, in which the comparison of interest was with saline. Data are presented graphically as means with variability presented as SEMs. Alpha level for all statistical tests was *p* ≤ 0.05. Effect sizes were calculated using the methods of Keppel and Wickens (2004).

### Drugs, Chemicals, and Reagents

Triazolam was purchased from Sigma-RBI (St. Louis, MO). XLi-JY-DMH and SH-TRI-108 were synthesized in the laboratory of JM Cook, Department of Chemistry, University of Wisconsin, Milwaukee (for synthesis, Hester and Von Voigtlander, 1979; Cook et al., 2003; Cook et al., 2006). Drugs were dissolved in small amounts of 95% ethanol if needed and then diluted to the desired concentrations in a 50% propylene glycol/50% saline solution. Other chemicals and reagents were obtained from the following sources: Dulbecco’s modified Eagle’s medium, l-glutamine, ß-mercaptoethanol, Penicillin G, streptomycin, and MEM from Invitrogen (Carlsbad, CA); fetal calf serum from Cambrex Corporation (East Rutherford, NJ) [3H]flunitrazepam from PerkinElmer Life Sciences (Boston, MA); and Tricain and trypsin inhibitor I-S from Sigma-Aldrich (St. Louis, MO).

### Molecular Docking Method

Ligand-protein interactions were analyzed by molecular docking using AutoDock Vina 1.5.6 (Trott and Olson, 2010). The Protein Data Bank (PDB) file of the CryoEM structure of the human full-length α1β3γ2L GABA_A_ receptor in complex with alprazolam (PDB: 6HUO) (Masiulis et al., 2019) was downloaded and prepared for docking by fixing missing bonds or atoms, adding polar hydrogens and assigning charges by AM1-BCC (Austin Model1 with bond charge correction), and removing water molecules. The protein was validated by first removing the bound ligand (alprazolam), and this was followed by docking it in the same binding site. The compounds were drawn and energy minimized in Chimera. A grid size of 16–16–16 Å of the 6HUO PDB structure was used, centered at coordinates 152.80 (x), 163.02 (y), and 161.14 (z). Illustrations of the three-dimensional models were generated using Chimera (Pettersen et al., 2004) and *Python* (Sanner, 1999). Dockings were performed with standard search parameters and poses were refined (the binding pose similar to alprazolam, which acts as a representative positive allosteric modulator, was selected). From the refined poses, the best score poses were selected for the analysis. Molecular docking was performed with triazolam, XLi-JY-DMH, and SH-TRI-108, in comparison with 8-substituted imidazodiazepines for which previous data from our laboratories have been published (Figure 1; Fischer et al., 2010; Duke et al., 2018): XHe-II-053 (8-ethynyl-6-phenyl-4*H*-2,5,10 b-triaza-benzo [*e*]azulene-3-carboxylic acid ethyl ester) and HZ-166 (8-ethynyl-6-(2ʹ-pyridine)-4*H*-2,5,10b-triaza-benzo [*e*]azulene-3-carboxylic acid ethyl ester).

## Results

### Binding Profiles at Multiple Subtypes of GABA_A_ Receptors

Triazolam potently and non-selectively displaced the binding of [^3^H]flunitrazepam at recombinant GABA_A_ receptors containing α1, α2, α3 and α5 subunits (Table 2). Similarly, XLi-JY-DMH non-selectively competed with [^3^H]flunitrazepam at all GABA_A_ receptors. Its affinity, however, was lower than that of triazolam at every receptor subtype, ranging from 4-fold lower at α5 subunit-containing receptors to 13-fold at α1 subunit-containing receptors. SH-TRI-108 also exhibited a non-selective binding profile. Additionally, its affinity was markedly lower than both triazolam and XLi-JY-DMH at all receptor subtypes (range compared to triazolam: 136-fold at α5 subunit-containing receptors to 475-fold at α1 subunit-containing receptors).

**TABLE 2:** Binding affinities of benzodiazepine receptor ligands at recombinant GABA_A_ receptors containing *a* subunits. Data are mean ± SD of K_i_ values, based on three independent experiments performed in triplicate.

	Binding affinity			
	K_i_ (nM)			
Drug	α1	α2	α3	α5
Triazolam	0.6 ± 0.3	0.8 ± 0.2	0.5 ± 0.1	0.9 ± 0.2
XLi-JY-DMH	7.7 ± 5.5	7.2 ± 3.0	4.2 ± 1.6	3.8 ± 1.0
SH-TRI-108	285.0 ± 182.0	248.0 ± 294.0	117.0 ± 26.0	122.0 ± 37.0

### Efficacy

**FIGURE 2:**
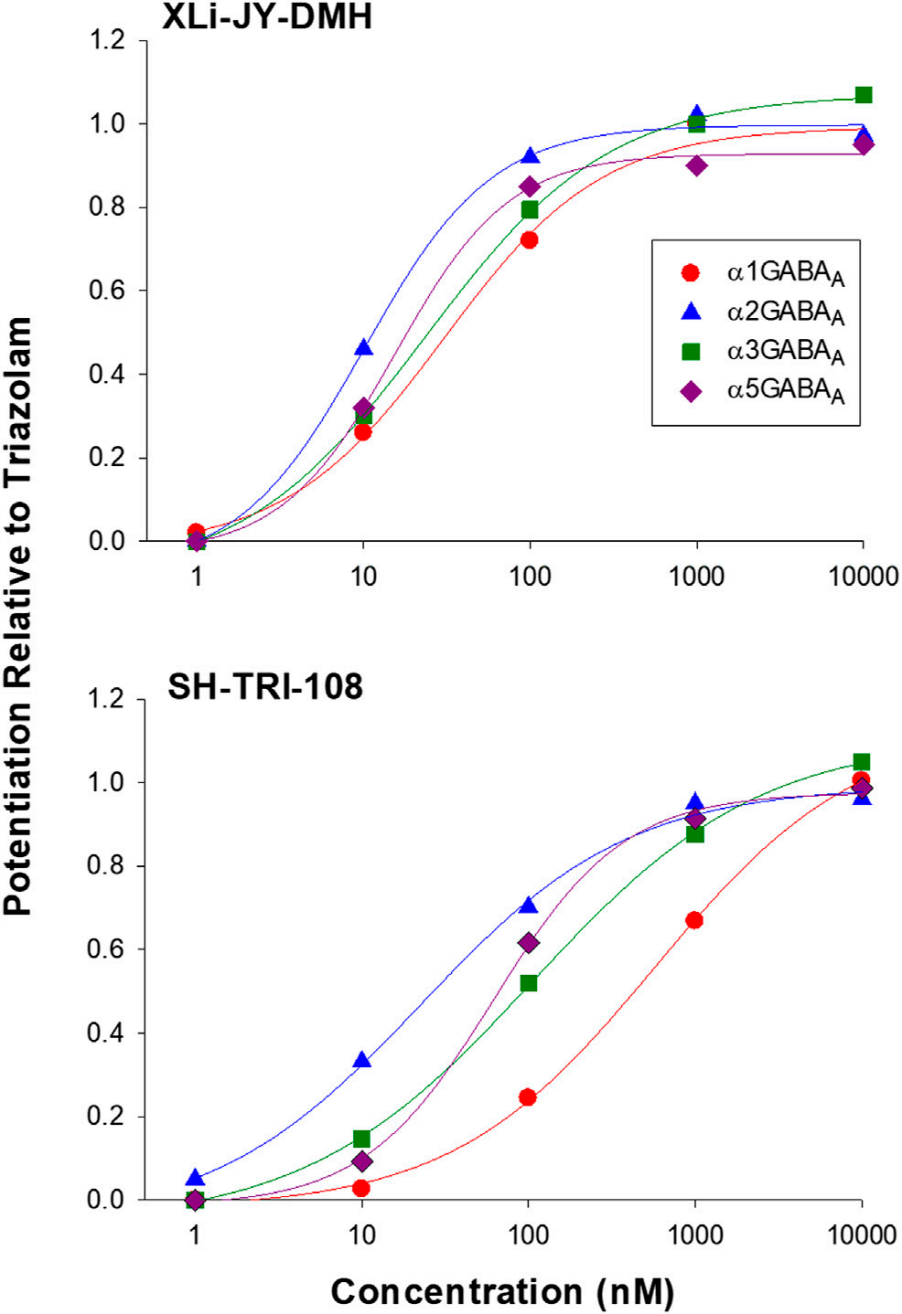
Modulation of recombinant αxGABA_A_ subtypes by 8-substituted triazolobenzodiazepines XLi-JY-DMH and SH-TRI-108. Data are expressed as percentage of triazolam effects at each concentration, based on modulation of the ability of GABA (EC_3_) to potentiate Cl^−^ currents measured via patch-clamp techniques. Each point is based on N = 3-5 replications.

In general, all three ligands exhibited robust and concentration-dependent enhancement of GABA-mediated Cl^−^ currents, as expected of positive allosteric modulators (Figure 2; Table 3). Triazolam, the parent compound, showed no differences in potency (EC_50_ values) among the subtypes, consistent with affinity estimates, and resulted in curves with Hill slopes no different from 1.0. The highest maximum stimulation from baseline occurred at α3GABA_A_ receptors (852.3%) and the lowest at α1GABA_A_ subtypes (461.6%), a pattern of effects observed previously for other conventional BZs (e.g., diazepam, Fischer et al., 2010). The analog XLi-JY-DMH resulted in a profile of effects very similar to triazolam, albeit this ligand was ∼2.5 to 4 fold less potent than triazolam, consistent with the results from binding affinity assays (Table 2). Similar to triazolam, XLi-JY-DMH also engendered maximum levels of stimulation ranging from 475.8% at α1GABA_A_ subtypes to 851.1% at α3GABA_A_ subtypes. Therefore, XLi-JY-DMH, similar to the parent compound triazolam, can be described as a full positive modulator with no evident subtype selectivity.

**TABLE 3:** Intrinsic efficacy and potency of benzodiazepine receptor ligands at recombinant GABA_A_ receptors containing αXβ3γ2 subunits, measured by patch-clamp electrophysiology.

	α1	α2	α3	α5
**Triazolam**				
EC_50_ (nM) (95% CI)	9.0 (7.2–11.3)	8.4 (5.5–12.6)	17.3 (14.3–20.7)	5.8 (4.9–16.9)
Hill slope	1.00 ± 0.08	1.07 ± 0.18	1.18 ± 0.09	1.03 ± 0.14
Max % effect	461.6 ± 7.0	581.0 ± 17.2	852.4 ± 12.3	529.6 ± 5.6
**XLi-JY-DMH**				
EC_50_ (nM) (95% CI)	35.2 (15.8–78.4)	20.7 (13.7–31.2)	47.0 (24.8–89.1)	14.9 (9.6–23.0)
Hill slope	0.96 ± 0.23	1.04 ± 0.15	1.07 ± 0.24	1.10 ± 0.22
Max % effect	475.8 ± 33.9	591.0 ± 21.0	851.1 ± 59.0	427.8 ± 14.2
**SH-TRI-108**				
EC_50_ (nM) (95% CI)	497.6 (373.3–663.4)	82.0 (64.1–104.9)	333.0 (225.5–491.5)	129.9 (90.1–187.3)
Hill slope	0.93 ± 0.08	0.99 ± 0.11	0.96 ± 0.12	0.94 ± 0.14
Max % effect	545.3 ± 16.4	598.3 ± 12.5	959.7 ± 38.9	525.5 ± 16.6

In contrast to triazolam and XLi-JY-DMH, SH-TRI-108 showed a modest degree of selectivity. Analyses of EC_50_ values showed no overlap in 95% CIs for α1GABA_A_ vs. α2GABA_A_ (∼6- fold difference) and α5GABA_A_ (∼4-fold difference) concentration-response functions (Table 3). At higher concentrations, however, the amount of potentiation across the subunits was similar to the results with triazolam, suggesting that SH-TRI-108 is a full modulator at all subtypes. It is worth noting that maximum effect of SH-TRI-108 at the α1GABA_A_ subtype was achieved only at the highest concentration of 10 μM, making this value an interpolation of maximal effects, in contrast to the concentration-effect functions at the other three subtypes.

Because the prototypical non-selective BZ triazolam actually has variations in efficacy and potency at different subtypes (e.g., functional selectivity at α3GABA_A_ receptors), we converted the entire concentration-response functions of XLi-JY-DMH and SH-TRI-108 to proportion of triazolam’s effects (Figure 2). Non-linear regression analysis was used to calculate potency and efficacy for the two analogs, which are shown in Table 4. As can be seen in the Table, the parameters of the logistic equation fit all curves with Hill slopes near 1.0. As with the raw data, the maximum effect (as proportion of triazolam) were essentially identical, with proportions close to 1.0 (i.e., equi-effective with triazolam). Potencies for both XLi-JY-DMH and SH-TRI-108 were lowest for α1GABA_A_ receptors. These differences for XLi-JY-DMH were modest and not significant, ranging from 1.3- to 3-fold. For SH-TRI-108, however, there was evidence of α1GABA_A_ *functional* selectivity, with a ∼25-fold, ∼5.4-fold, and ∼9.2-fold difference in potency vs. α2GABA_A_, α3GABA_A_, and α5GABA_A_ subtypes, respectively. Although statistically significant (*p*’s < 0.05), the key question becomes to what extent does this *in vitro* functional selectivity for SH-TRI-108 translate to behaviorally meaningful selectivity in anxiolytic-like and sedative-motor effects.

**TABLE 4:** Intrinsic efficacy and potency of benzodiazepine receptor ligands at recombinant GABA_A_ receptors containing αXβ3γ2 subunits based on all data converted **to proportion of triazolam**.

	α1	α2	α3	α5
**XLi-JY-DMH**				
EC_50_ (nM)	30.3 ± 12.2	10.1 ± 2.1	23.3 ± 2.8	15.5 ± 2.3
Hill slope	0.91 ± 0.23	1.14 ± 0.15	0.88 ± 0.07	1.21 ± 0.25
Max effect (proportion of triazolam)	1.01 ± 0.13	1.07 ± 0.10	1.16 ± 0.05	0.96 ± 0.05
**SH-TRI-108**				
EC_50_ (nM)	574.1 ± 95.0	22.6 ± 8.4	105.9 ± 9.5	61.8 ± 8.3
Hill slope	0.83 ± 0.08	0.89 ± 0.16	0.86 ± 0.09	1.11 ± 0.16
Max effect (proportion of triazolam)	1.16 ± 0.06	1.05 ± 0.13	1.17 ± 0.04	0.99 ± 0.04

### Anxiolytic-like Effects

**FIGURE 3:**
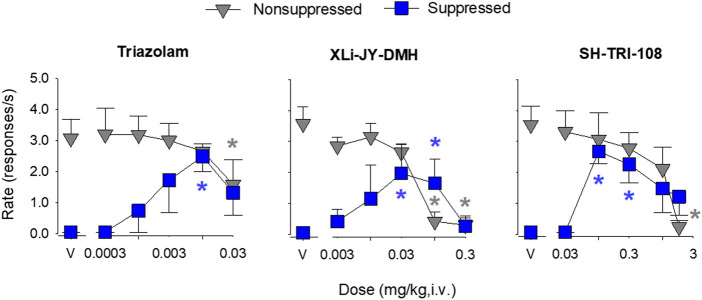
Effects of benzodiazepine receptor ligands in rhesus monkeys responding under a two-component multiple schedule in which responding was maintained under a fixed-ratio schedule of food delivery in the absence (non-suppressed responding) and presence of response-contingent electric shock (suppressed responding). Data are mean ± SEM for N = 3 rhesus monkeys. Blue asterisks (*): *p* < 0.05 vs. vehicle for suppressed responding (blue squares); Gray asterisks (*) *p* < 0.05 vs. vehicle for non-suppressed responding (gray triangles), Fisher’s method.

The rhesus conflict model can differentiate α1GABA_A_-preferring compounds from those with selectivity for α2/3GABA_A_ receptors (e.g., Fischer et al., 2010) with the former compounds ineffective in increasing rates of suppressed responding, whereas the latter compounds increase rates of suppressed responding, but have no effects on non-suppressed responding (Rowlett et al., 2005; 2006). During training sessions, mean rates of responding in non-suppressed (food only) components were between 3.0 and 4.0 responses/s, whereas rates of responding in suppressed (food + shock) components were at or near zero. During tests with drug vehicles, rates of responding in the non-suppressed and suppressed components showed a similar pattern as during training (i.e., relatively high response rates in the absence of shock, little or no responding when shock was present; Figure 3, gray vs. blue symbols above “V”).

Intravenous administration of triazolam engendered a characteristic increase in the rates of suppressed responding at low-to-intermediate doses and attenuated the rates of non-suppressed responding at higher doses (Figure 3, left panel; see Table 5 for results). The effects of triazolam were dose-dependent, with a cumulative dose of 0.01 mg/kg engendering a reliable increase in the mean rate of suppressed responding compared to the response rate after the administration of vehicle (*p* < 0.05, Fisher’s method). The highest dose of triazolam (0.03 mg/kg) reliably decreased the mean rate of non-suppressed responding compared to vehicle (*p* < 0.05). Comparing the minimum effective dose (MED) to alter suppressed responding (0.01 mg/kg) with the MED to alter non-suppressed responding (0.03 mg/kg), triazolam was 3-fold more potent at increasing suppressed responding vs. decreasing non-suppressed responding.

**TABLE 5:** Summary of omnibus tests of significance (repeated measures analyses of variance) for the behavioral experiments with triazolobenzodiazepines.

Study	Effect	Drug/Compound	F Value	Degrees of freedom (numerator, denominator)	*p* Value	Effect size (*r* ^2^)*
Conflict						
	Non-suppressed	Triazolam	15.39	5, 10	<0.001	0.885
	Suppressed	Triazolam	20.61	5, 10	<0.0001	0.912
	Non-suppressed	XLi-JY-DMH	19.98	5, 10	<0.0001	0.910
	Suppressed	XLi-JY-DMH	17.11	5, 10	<0.001	0.873
	Non-suppressed	SH-TRI-108	21.99	5, 10	<0.0001	0.919
	Suppressed	SH-TRI-108	14.57	5, 10	<0.001	0.867
Observable Effects						
	Locomotion	XLi-JY-DMH	2.78	6, 18	0.043	0.514
	Environment-Directed	XLi-JY-DMH	2.80	6, 18	0.042	0.519
	Rest Posture	XLi-JY-DMH	2.71	6, 18	0.047	0.495
	Procumbent	XLi-JY-DMH	6.54	6, 18	<0.001	0.972
	Ataxia	XLi-JY-DMH	4.27	6, 18	0.008	0.814
	Resistance	XLi-JY-DMH	27.67	6, 18	<0.001	0.989
	Locomotion	SH-TRI-108	7.78	4, 12	0.002	0.950
	Environment-Directed	SH-TRI-108	4.64	4, 12	0.017	0.712
	Rest Posture	SH-TRI-108	10.69	4, 12	<0.001	0.991
	Procumbent	SH-TRI-108	14.48	4, 12	<0.001	0.999
	Ataxia	SH-TRI-108	32.20	4, 12	<0.001	0.999
	Resistance	SH-TRI-108	48.21	4, 12	<0.001	0.999
	Pellet Consumption	Triazolam	7.64	5, 18	<0.001	0.914
		XLi-JY-DMH	5.96	5, 18	<0.001	0.807
		SH-TRI-108	6.33	5, 18	<0.001	0.896

* Effect size calculated by the method of Keppel and Wickens (2004).

In general, XLi-JY-DMH produced effects similar to triazolam (Figure 3, middle panel; see Table 5 for results). XLi-JY-DMH dose-dependently increased rates of suppressed responding over the dose range of 0.003–0.1 mg/kg. Response rates after cumulative doses of 0.03 and 0.1 mg/kg were reliably different from vehicle (*p*’s < 0.05). XLi-JY-DMH dose-dependently decreased rates of responding in the non-suppressed components, with the two highest doses (0.1 and 0.3 mg/kg) virtually eliminating responding (*p*’s < 0.05). Comparing the MED to alter suppressed responding (0.03 mg/kg) with the MED to alter non-suppressed responding (0.1 mg/kg), XLi-JY-DMH also was 3-fold more potent at increasing suppressed responding vs. decreasing non-suppressed responding.

Like triazolam and XLi-JY-DMH, SH-TRI-108 dose-dependently increased rates of suppressed responding, with response rates after cumulative doses of 0.1 and 0.3 mg/kg reliably different from vehicle (Figure 3, right panel; *p*’s < 0.05; see Table 5 for results). Rates of responding in the non-suppressed components were dose-dependently decreased, and response rates after the highest dose of SH-TRI-108 were significantly lower than rates after vehicle (*p* < 0.05). Although the effects of SH-TRI-108 were qualitatively similar to the other benzodiazepine receptor ligands, comparison of the MED to alter suppressed responding (0.1 mg/kg) with the MED to alter non-suppressed responding (1.8 mg/kg) showed that SH-TRI-108 was 18-fold more potent at increasing suppressed responding vs. decreasing non-suppressed responding.

### Observable Effects

**FIGURE 4:**
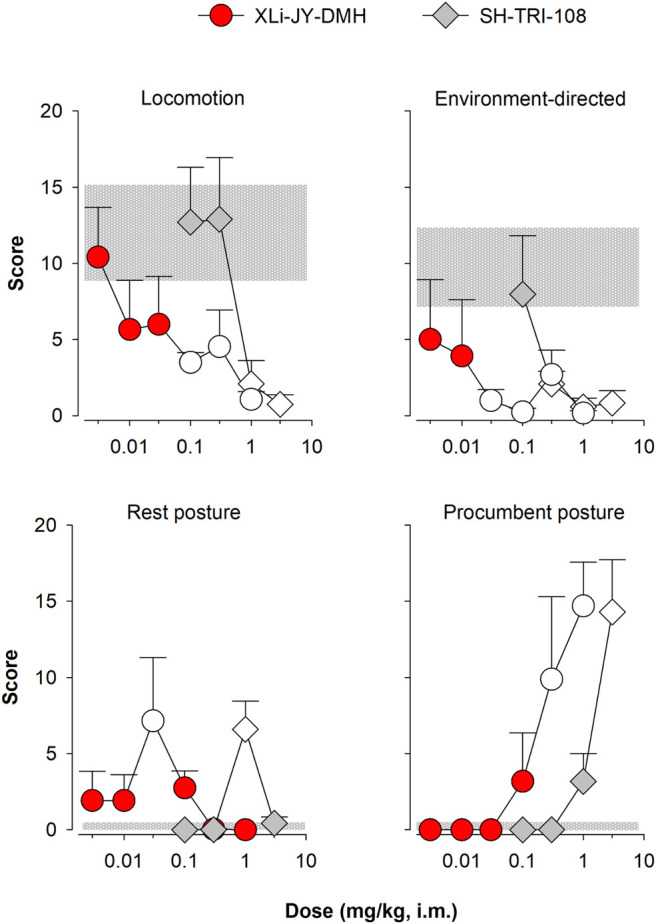
Observable behavior following i. m. injections of XLi-JY-DMH and SH-TRI-108 in squirrel monkeys (N = 4). Data are mean modified frequency score (+/- SEM) for four behaviors altered significantly by treatments (see Table 1 for definitions). Symbols filled with white represent significant differences from vehicle, represented as gray horizontal bars (lower and upper SEMs), Fisher’s method.

Our squirrel monkey observation procedures also can differentiate α1GABA_A_-preferring ligands from non-selective ligands under certain conditions (Platt et al., 2002; Rowlett et al., 2005; Licata et al., 2009). Of note, triazolam was evaluated in both Platt et al. (2002) and Licata et al. (2009), and those previous data were used as comparators to the present findings with XLi-JY-DMH and SH-TRI-108. In the present study, the average frequency of self-directed behaviors (grooming, scratching) was low following saline administration and was not systematically affected by administration of any drug (data not shown). The levels of locomotion and environment-directed behavior (object exploration and foraging) at low doses did not differ from that observed on control days (Figure 4, top panels, compare points with horizontal gray bar; note that results are summarized in Table 5). However, at intermediate-to-high doses (≥0.03), XLi-JY-DMH reduced these behaviors (*p* < 0.05 in both cases). A single intermediate dose of XLi-JY-DMH (0.03 mg/kg) also engendered reliable increases in rest posture (Figure 4, bottom left panel; *p* = 0.05). The highest doses of XLi-JY-DMH (≥0.3 mg/kg) reliably increased procumbent posture (Figure 4, bottom right panel; 0.3 mg/kg: *p* < 0.05; 1.0 mg/kg: *p* < 0.001).

SH-TRI-108 engendered a similar pattern of effects on observable behavior. SH-TRI-108 reduced overall activity at higher doses (≥0.3 mg/kg), inducing significant decreases in both locomotion and environment-directed behavior (*p* < 0.05 for both behaviors; Figure 4, top panels). Concomitant with the decrease in these active behaviors, SH-TRI-108 induced significant increases in rest posture (1.0 mg/kg) and procumbent posture (3.0 mg/kg; Figure 4, bottom panels; rest posture: *p* < 0.001; procumbent posture: *p* < 0.001 in both cases).

Based on our previous research (e.g., Rowlett et al., 2005), one prediction based on *in vitro* activity at α1GABA_A_ subtypes is significant procumbent posture compared to other behaviors. Similar to triazolam previously (Platt et al., 2002), both XLi-JY-DMH and SH-TRI-108 engendered these behaviors. Because of the differences in GABA-mediated current potencies, we assessed the extent to which XLi-JY-DMH and SH-TRI-108 differed among the four behavioral effects induced by each compound. Comparison of MEDs showed that SH-TRI-108 was 10-fold less potent than XLi-JY-DMH for decreases in locomotion and environment-directed behavior, and increases in procumbent posture, with a 33-fold difference in MEDs for increases in rest posture (Table 6).

**TABLE 6:** Potencies determined by minimum effective dose (MED, mg/kg) for induction of observable behavioral effects and from hands-on assessment following triazolam and triazolobenzodiazepines in squirrel monkeys (N = 4).

Ligand	Locomotion	Environment-Directed	Rest Posture	Procumbent Posture	Food Consumption Increase	Ataxia	Muscle Resistance
Triazolam	0.03*	0.1*	0.1*	0.1*	0.03	0.1*	0.1*
XLi-JY-DMH	0.1	0.03	0.03	0.3	1.0	0.1	0.03
SH-TRI-108	1.0	0.3	1.0	3.0	1.0	1.0	0.3

* Triazolam data were derived from previously published reports using the same methodology (Platt et al., 2002; Duke et al., 2006; Licata et al., 2009).

**FIGURE 5:**
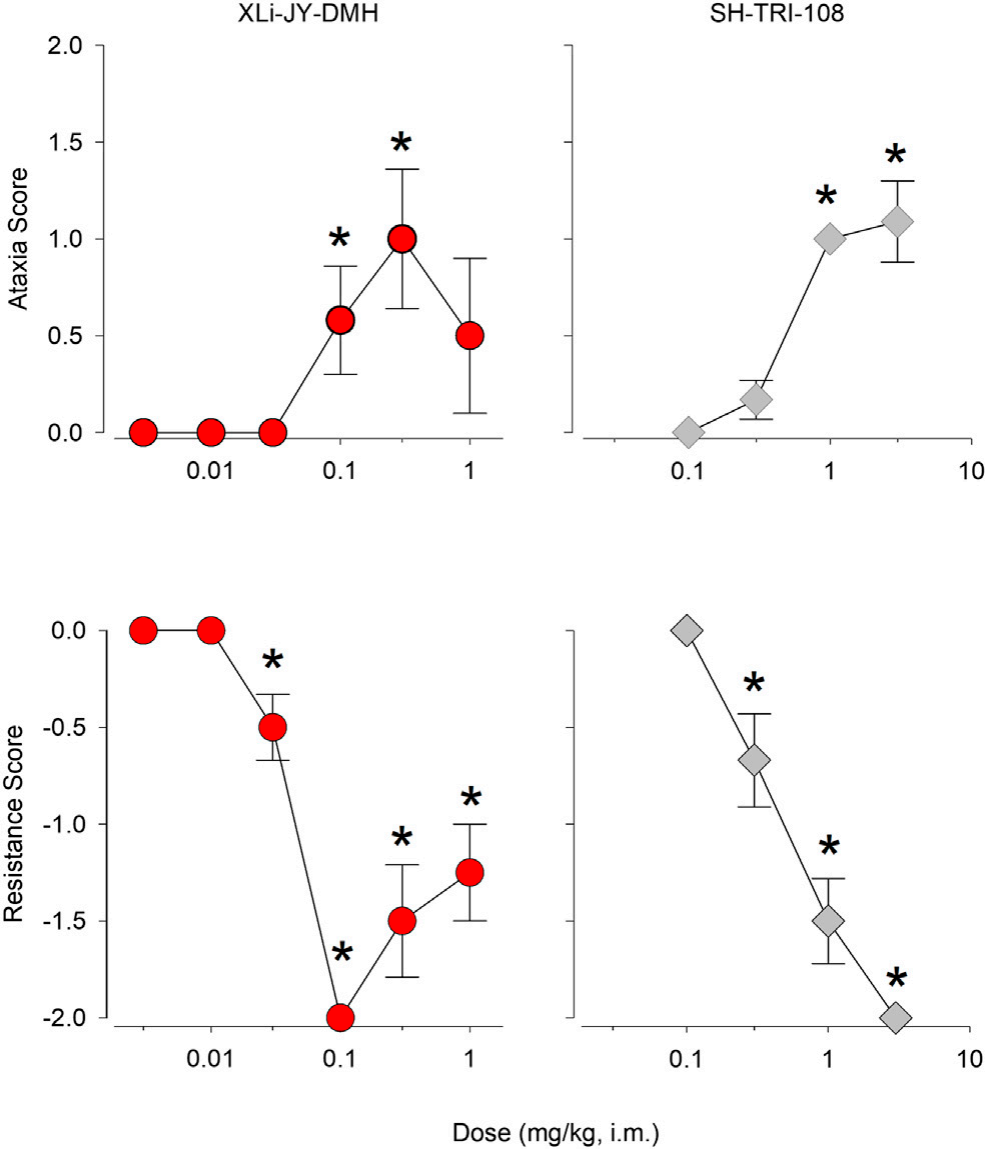
Experimenter-assessed ataxia and muscle resistance (relaxant) scores following i. m. injections of XLi-JY-DMH and SH-TRI-108 in squirrel monkeys (N = 4). Data are mean ± SEM. * *p* < 0.05 vs. vehicle, Fisher’s method.

Results from hands-on assessments of ataxia and muscle resistance are depicted in Figure 5 (see Table 5 for results). For XLi-JY-DMH, the dose-response function for mean ataxia scores was biphasic, with the 0.1 and 0.3 mg/kg doses engendering significant increases from vehicle (*p* < 0.05). In contrast, SH-TRI-108 showed a monotonic dose-response function for mean ataxia scores, with doses of 1.0 and 3.0 mg/kg significantly greater than vehicle (*p* < 0.05). Both compounds also significantly decreased mean resistance scores (Figure 5, bottom panels), with XLi-JY-DMH again showing a biphasic dose-response function, in which doses above 0.1 mg/kg tended to return to resistance scores of zero (although still significantly below vehicle, *p* < 0.05). SH-TRI-108, in contrast, demonstrated a dose-dependent decrease in mean resistance scores, with the dose of 0.3 mg/kg and above significantly lower than vehicle (*p* < 0.05). Comparison of MED values for both compounds between the two measures revealed that decreases in muscle resistance occurred at a lower dose, i.e., was a more potent effect, than ataxia for both compounds, although the difference was relatively modest (3.3-fold).

**FIGURE 6:**
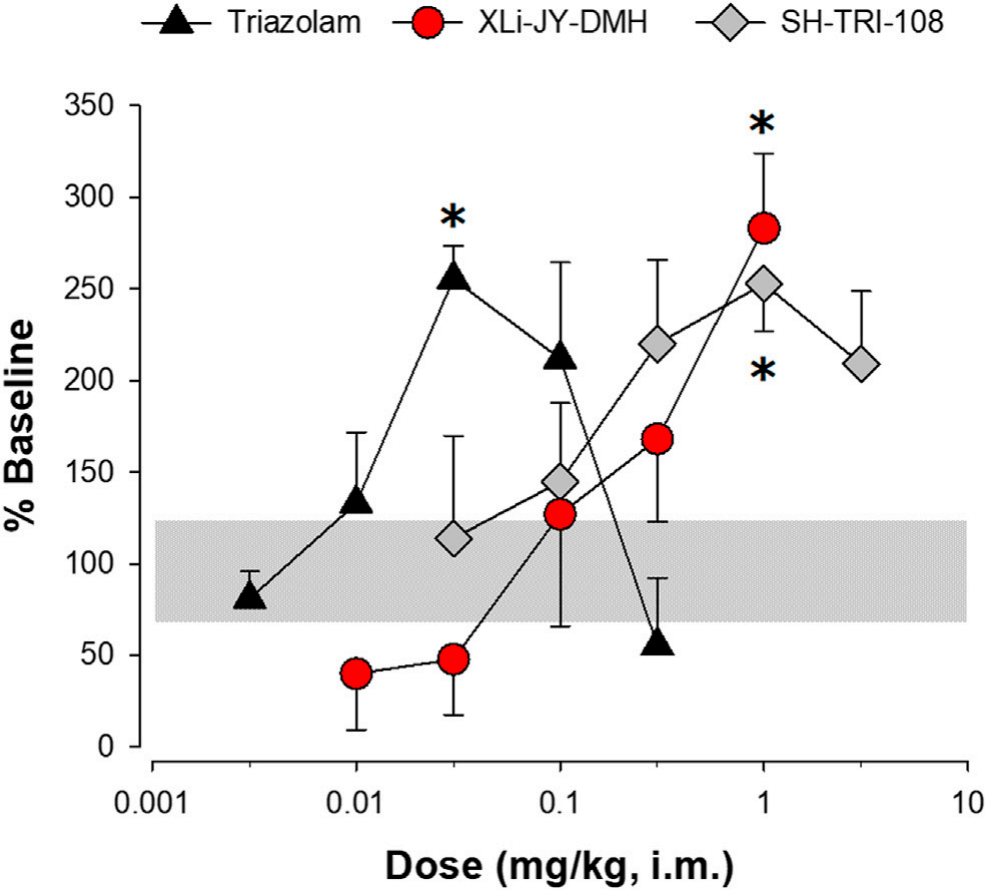
Sucrose pellet consumption by squirrel monkeys (N = 4) following i. m. injections of triazolam, XLi-JY-DMH, or SH-TRI-108. Data are percent of baseline pellet consumption, the horizontal gray bar represents lower and upper SEMs for vehicle treatment. * *p* < 0.05 vs. vehicle, Fisher’s method.

Consistent with our previous research (Duke et al., 2006), sucrose pellet consumption was increased to approximately 250–300% of baseline values by triazolam, XLi-JY-DMH, and SH-TRI-108 (Figure 6; see Table 5 for results). Compared with vehicle tests, the 0.03 mg/kg dose of triazolam and 1.0 mg/kg for both triazolobenzodiazepine compounds resulted in significantly increase levels of sucrose pellet consumption. A noteworthy observation is that doses above the 0.03 mg/kg of triazolam and 1.0 mg/kg of SH-TRI-108 did not significantly alter sucrose pellet consumption, suggesting a biphasic function, whereas XLi-JY-DMH engendered a monotonic increase in pellet consumption over the dose range tested.

Summaries of the potencies for triazolam and the triazolobenzodiazepine effects on observed behavior are shown in Table 6, based on minimum effective doses (MEDs). For most behaviors, triazolam and XLi-JY-DMH were essentially equipotent, varying by no more than plus/minus 3-fold, whereas SH-TRI-108 was generally less potent, ranging from 3- to 33-fold higher MED values. The only variant from this pattern was XLi-JY-DMH being 33-fold less potent than triazolam and equipotent with SH-TRI-108 in inducing increased sucrose pellet consumption.

### Molecular Docking

**FIGURE 7:**
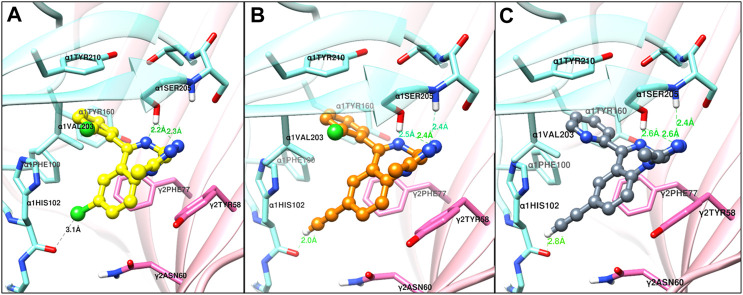
The docked confirmations of triazolam (yellow), XLi-JY-DMH (orange) and SH-TRI-108 (gray) in the complex with the α1β3γ2L GABA_A_ receptor 6HUO at the α^+^γ^−^ interface benzodiazepine binding site [α1 (aquamarine) and γ2 (orchid)], dashed lines indicate hydrogen bonds (green) and halogen bond (black).

**FIGURE 8:**
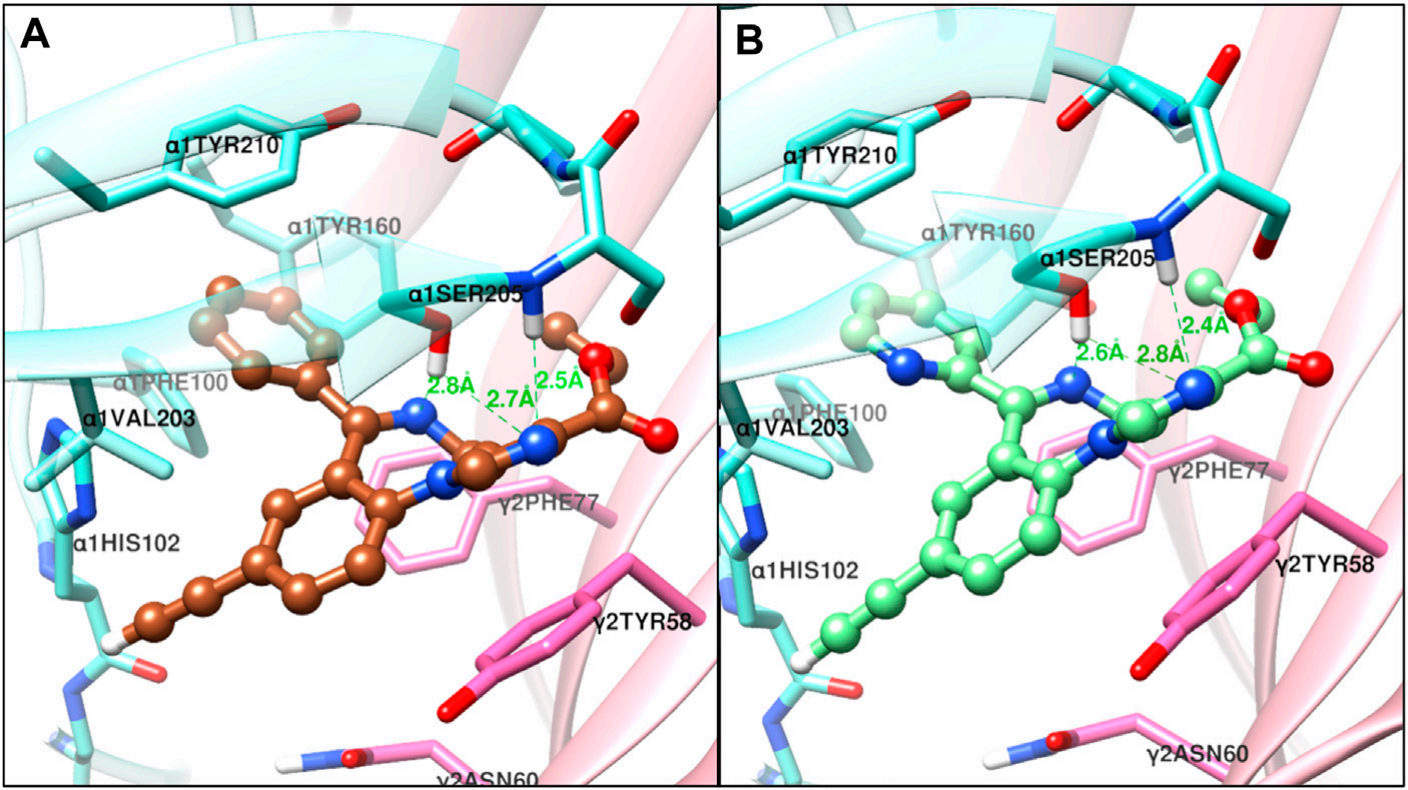
The docked confirmations of XHe-II-053 (sienna) and HZ-166 (light green) in the complex with the α1β3γ2L GABA_A_ receptor 6HUO at the α^+^γ^−^ interface benzodiazepine binding site [α1 (aquamarine) and γ2 (orchid)], dashed lines indicate hydrogen bonds (green) and halogen bond (black).

**FIGURE 9:**
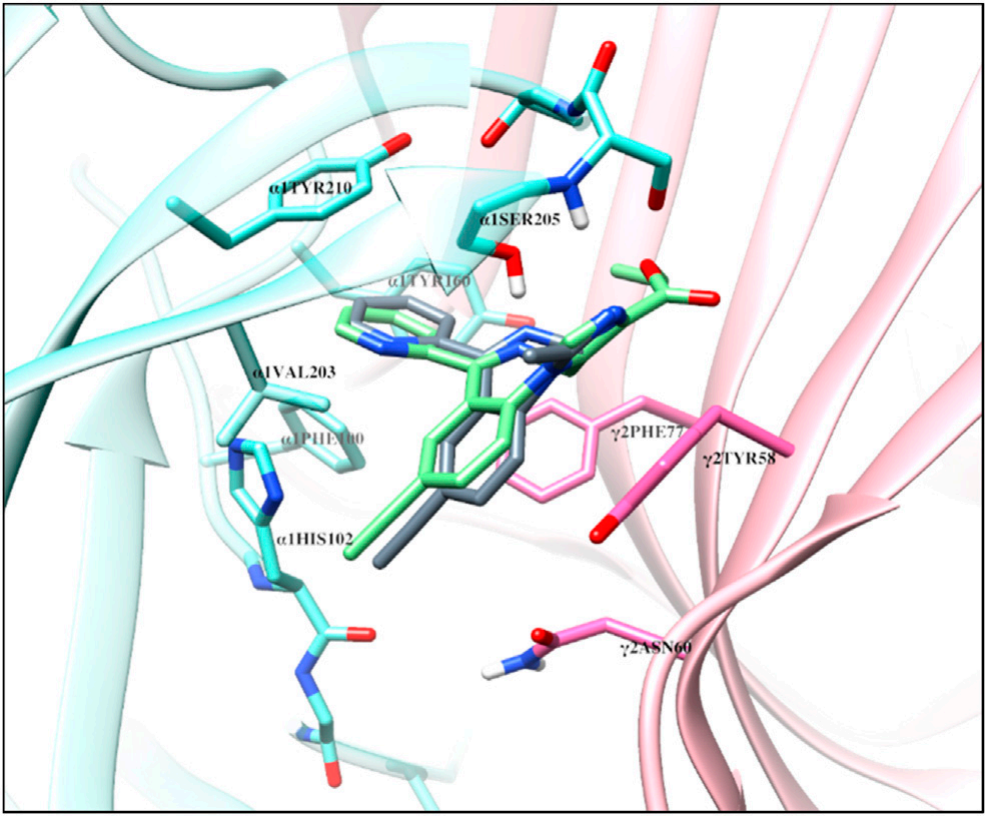
The overlap of the docked confirmation of SH-TRI-108 and HZ-166 in the complex with α1β3γ2L GABA_A_ receptor 6HUO at the α^+^γ^−^ interface of the benzodiazepine binding site [α1 (aquamarine) and γ2 (orchid)].

The structural docking studies were conducted to elucidate the unique molecular interactions of the 8-ethynyl triazolobenzodiazepines (XLi-JY-DMH, SH-TRI-108) and previously published 8-ethynyl imidazodiazepines (XHe-II-053 and HZ-166) with the α1 subunit-containing GABA_A_ receptor. Described both below and in Figures 7–9 are the interactions between the receptor binding site and the ligands. The Figures show the α+γ-binding interface of the α1GABA_A_ receptor, with the α1 interface in aquamarine and the γ2 interface in orchid, with the ligands posed to the C-loop of the α1 subunit, a component of the receptor well-established as critical to ligand-mediated binding and gating (cf. Terejko et al., 2020). The triazolam structure was docked in a similar conformation as bound alprazolam in the CryoER structure (6HUO). The chlorine atom at the C8 position in triazolam forms a halogen bond with the carbonyl oxygen of the α1His102 amino acid backbone which is absent in 8-substituted imidazodiazepines (compare Figure 7A with Figure 8). It is clear that the interactions between the C-loop and the triazolobenzodiazepines, i.e., stabilization of the C-loop in the open conformation, are stronger than the interactions between the C-loop and the imidazodiazepines (compare Figures 7, 8). This likely is the reason that triazolam and the triazolobenzodiazepines are more prominently sedating ligands, whereas the imidazodiazepines showed no appreciable or reduced benzodiazepine-like sedation (Rivas et al., 2009; Fischer et al., 2010; Di Lio et al., 2011; Paul et al., 2014; Duke et al., 2018; Tudeau et al., 2020). Illustrated in Figures 7, 8 are the docking poses of ligands 1–5. Docking poses of 8-ethynyl triazolobenzodiazepines indicates that the triazole ring system occupies the same location as the ligand alprazolam bound to the receptor (PDB 6HUO). The triazole ring stabilizes the C-loop in the open position via the hydrogen bonds with the side chain hydroxyl group and the backbone amide nitrogen of α1Serine205. The imine nitrogen of ligands 2 and 3 forms a hydrogen bond with the side chain of α1Serine205. The pendant phenyl rings of all compounds are packed in an aromatic box formed by α1Tyr210, α1Tyr160, α1Phe100, α1His102, and γ2Phe77. The terminal hydrogen of acetylene in triazolobenzodiazepines forms a weak hydrogen bond (distance 2 and 2.8 Å, respectively) with the carboxyl oxygen of the α1His102 backbone (Figures 7B,C). These interactions help to stabilize the ion channel C-loop in the open position permitting chlorine ions to flow through the channel. In contrast to 8-ethynyl triazolobenzodiazepines, the terminal hydrogen of the acetylene in imidazodiazepines (ligands 4–5) does not form a hydrogen bond with the carboxyl oxygen of the α1His102 backbone. The imine nitrogen and imidazole ring nitrogen of imidazodiazepines do form hydrogen bonds with the α1Serine205 side chain but with longer (weaker) distances than that of triazolobenzodiazepines (compare Figures 7, 8). Examination of the overlay docking poses of SH-TRI-108 and HZ-166 shows a slightly different orientation of the acetylene moiety and pendant phenyl ring (Figure 9). The overall docking scores were in the order of (strongest to weakest binding energy) triazolam > XLi-JY-DMH > SH-TRI-108>XHe-II-053>HZ-166 (see Table 7).

**TABLE 7:** Binding energy (α1GABA_A_ subtype) estimated by autodock vina, compared with anti-conflict potency.

Compound	Docking score^a^ (kCal/mole)	Anti-conflict potency^b^ (MED_ns_/MED_s_)
Triazolam	−11.1	3
XLi-JY-DMH	−10.8	3
SH-TRI-108	−10.1	18
XHe-II-053	−9.3	NE (>10)^c^
HZ-166	−8.9	NE (>10)^c^

^a^ Triazolam binds more robustly to the α1β3γ2 binding site than SH-TRI-108 by 1 kCal/mole, thus defining an energy requirement for separation of anti-conflict from rate-suppressing (sedating) effects.

^b^ MED_ns_, minimum effective dose to attenuate responding maintained by food with no shock presentation (“non-suppressed responding”) in the rhesus conflict model. MED_s_, minimum effective dose to increase responding maintained by food but attenuated by contingent shock presentation (“suppressed responding”) in the rhesus conflict model.

^c^ NE, no effect; from Fischer et al. (2010).

## Discussion

Since the introduction of chlordiazepoxide and diazepam in the 1960’s, BZ-type drugs have become important psychiatric tools for the management of anxiety and sleep disorders. Over the past two decades, intense interest in exploiting the BZ-sensitive GABA_A_ receptor subtypes has resulted in clinical candidates that lack some of the deleterious side effects associated with this class of drugs (for review, see Maramai et al., 2020). A recent unique application of preclinical discovery and development are bioisostere 8-ethynyl-imidazodiazepines, which along with reduced sedative effects have shown clear promise as improved anticonvulsants for the treatment of epilepsy (e.g., Witkin et al., 2020). Here, we examined an additional series of 8-ethynyl analogs of triazolam (8-ethynyl-triazolobenzodiazepines), which take advantage of triazolam’s relatively high degree of efficacy and desirable overall potency.

The compounds studied in our program, XLi-JY-DMH and SH-TRI-108, retained the relatively high intrinsic efficacy and potency of triazolam in *vitro* evaluations. For both the triazobenzodiazepine and imidazodiazepine series, the 8-ethynyl modification appears to result in some loss of potency, although there is an overall shift in efficacy profiles to differentiation between α1GABA_A_ and α2/α3GABA_A_ subtypes (Poe et al., 2016; Sieghart and Savić, 2018). For example, the imidazodiazepine KRM-II-81 (5-(8-ethynyl-6-(pyridin-2-yl)-4H-benzo [f]imidazo[1,5-a][1,4]diazepin-3-yl)oxazole) demonstrated a greater degree of intrinsic efficacy at α2/α3GABA_A_ subtypes relative to α1GABA_A_ subtypes (e.g., Witkin et al., 2017; Witkin et al., 2020), although at higher concentrations this and related compounds do show at least partial intrinsic efficacy at the latter subtypes (leading to the suggested descriptor of “α2/α3GABA_A_ subtype-preferring compounds”; Maramai et al., 2020).

As discussed by Sieghart and Savić (2018), selectivity of a compound should be conceptualized as an interaction of potency, efficacy, and target engagement. In this regard, it is feasible that a compound may have substantial intrinsic efficacy at a subtype, but the potency at this subtype is low enough that sufficient CNS concentrations cannot be attained following peripheral administration (Sieghart and Savić, 2018). For the present study, such a scenario was postulated for SH-TRI-108. For example, at a concentration of 100 nM, positive modulation of 24% relative to triazolam was obtained for α1GABA_A_ subtypes, whereas the positive modulation at the other three subtypes was 52% or above. This differentiation was most notable for α2GABA_A_ receptors, for which the positive modulation of GABA-mediated Cl^−^ currents was 70% relative to triazolam. This degree of functional selectivity has resulted in “anxio-selective” effects for other compounds (for review, see Sieghart and Savić, 2018; Maramai et al., 2020). At higher concentrations, however, functional selectivity was not evident, suggesting that SH-TRI-108 would show behavioral selectivity over a narrower range of doses than a compound deemed functionally selective based on an absence of intrinsic efficacy at α1GABA_A_ receptors.

To determine if the level of selectivity exhibited by SH-TRI-108 *in vitro* translated to a unique behavioral profile *in vivo*, we conducted behavioral pharmacology studies in monkeys using the approach described by Rowlett et al. (2005). Although overall differences in potency among triazolam, XLi-JY-DMH, and SH-TRI-108 were observed, there was little to distinguish the compounds with respect to selective anxiolytic-like effects. In a rhesus monkey conflict model of anxiolytic-like effects, SH-TRI-108 showed a greater degree of separation (18-fold) between suppressed and non-suppressed responding (i.e., anti-conflict effect) compared with triazolam and XLi-JY-DMH (both 3-fold), this 18-fold difference is in some instances smaller than observed for other known anxiolytic, yet sedative, drugs in this same procedure (Rowlett et al., 2005; Rowlett et al., 2006). Similarly, SH-TRI-108 induced observable effects and increases in food consumption in squirrel monkeys that were very similar to those observed with triazolam and other BZs, but not compounds with α2/3/5GABA_A_ functional selectivity (e.g., Platt et al., 2002; Rowlett et al., 2005; Duke et al., 2006). In general, we conclude that the degree of selectivity (25-fold α2GABA_A_ vs. α1GABA_A_ subtypes) shown by SH-TRI-108 likely is insufficient to result in an anxio-selective profile for a novel compound.

Emerging evidence has strongly implicated the α1GABA_A_ receptor in mediating sedative-motor effects, but not anxiolytic effects, of benzodiazepines (for review, see Engin et al., 2018). Consistent with this observation, our previous work with imidazodiazepines suggests that these compounds, in contrast to the triazolobenzodiazepines, have lower efficacy at α1GABA_A_ subtypes compared with α2/3GABA_A_ subtypes, and importantly, a lower degree of sedation and motor impairments (cf., the present study; Fischer et al., 2010; Duke et al., 2018). Therefore, we hypothesized that binding of the triazolobenzodiazepines would be more robust at the level of the binding site than imidazodiazepines, and conducted structural modeling to evaluate this hypothesis. At the benzodiazepine binding site on α1GABA_A_ receptors, the interactions between triazolam and the two triazolobenzodiazepine analogs with the C-loop were similar, yet stronger than those of the two imidazodiazepines XHe-II-053 and HZ-166. C-loop stabilization in the open conformation is thought to mediate GABA-modulated opening of the Cl^−^ channel (Masiulis et al., 2019). The docking scores (Table 7) indicate that binding energies of 8-ethynyl imidazodiazepine ligands were weaker when compared to 8-ethynyl triazolobenzodiazepines. This is due to the lack of a hydrogen bond with the terminal hydrogen of the acetylene moiety and longer hydrogen bond distance with the C-loop amino acid α1Serine205. For comparison with *in vivo* results, we also included results from our conflict model in Table 7. These data represent a fold-difference in the MED to produce an anxiolytic-like effect vs. suppression of rate, the latter correlated with mild-to-moderate sedation using observation procedures (Duke et al., 2018), with the higher number indicating more separation between doses that engender anxiolytic-like effects vs. sedation. This separation was similar between triazolam and XLi-JY-DMH, in which the docking scores were closely aligned, yet higher for SH-TRI-108, which has a lower docking score than the other two ligands. Strikingly, the compounds XHe-II-053 and HZ-166 had no measurable effect on rates of responding, corresponding to the two lowest docking scores. Indeed, HZ-166 had no moderate-to-deep sedation or ataxia, as measured by observation techniques in rhesus monkeys, up a dose of 30 mg/kg, i. v. (it should be noted that a mild form of sedation, referred to as “rest/sleep posture”, is observed with all α2/3/5GABA_A_-selective compounds). Collectively, these data suggest that a BZ-type ligand’s sedative profile may be predicted based on C-loop binding energy of the compound with the α1GABA_A_ receptor binding site.

There are many factors to consider when determining the extent to which differences in subtype affinities and efficacies might predict behavioral properties of compounds, not the least of which are factors such as absorption, distribution, brain penetrability, etc. Pharmacokinetic variables were less likely to account for behavioral effects given the triazolam scaffold of the two compounds, although they nevertheless should be considered. One curious property of these compounds was the lack of any appreciable selectivity among subtypes in terms of receptor binding affinity. Therefore, the functional selectivity shown by SH-TRI-108 likely was not determined by differences in binding affinity—an unexpected finding for which we do not have additional information at this point. Overall, the conclusions of α1GABA_A_ C-loop binding energy predicting sedative effects require further confirmation with more compounds, including structurally diverse ones, as well as compounds with differing selectivity profiles. Moreover, the interplay with other subtypes (α2GABA_A_, α3GABA_A_, α5GABA_A_) awaits further modeling studies.

In conclusion, at the most basic level the triazolobenzodiazines assessed here did not possess behavioral selectivity, and therefore do not warrant further development as possible treatments. However, these results provide guidance for the degree of selective efficacy required to obtain *in vitro* to translate into clinically relevant separation of anxiolytic-like and sedative motor effects in preclinical studies. Specifically, *in silico* determination of docking scores at the α1 subunit C-loop of less than 10 kCal/mole are predicted to result in compounds with fewer sedative-motor side effects. In context, bioisostere 8-ethynyl imidazodiazepines have been shown consistently to have reduced sedative effects, yet retain preclinical effects predictive of anxiolysis, as well as anti-epilepsy and anti-nociceptive properties (Fischer et al., 2010; Duke et al., 2018; Witkin et al., 2017; 2018; 2020), suggesting this strategy of compound development with imidazodiazepines to be the more viable approach.

## Data Availability

The original contributions presented in the study are included in the article, further inquiries can be directed to the corresponding author.
